# Predicting the risk of end-stage renal disease in the population-based setting: a retrospective case-control study

**DOI:** 10.1186/1471-2369-12-17

**Published:** 2011-05-05

**Authors:** Eric S Johnson, David H Smith, Micah L Thorp, Xiuhai Yang, Juhaeri Juhaeri

**Affiliations:** 1 The Center for Health Research, Kaiser Permanente Northwest, Portland, Oregon, USA; 2 Department of Nephrology, Kaiser Permanente Northwest, Portland, Oregon, USA; 3 Sanofi-Aventis, Bridgewater, New Jersey, USA

## Abstract

**Background:**

Previous studies of predictors of end-stage renal disease (ESRD) have limitations: (1) some focused on patients with clinically recognized chronic kidney disease (CKD); (2) others identified population-based patients who developed ESRD, but lacked earlier baseline clinical measures to predict ESRD. Our study was designed to address these limitations and to identify the strength and precision of characteristics that might predict ESRD pragmatically for decision-makers--as measured by the onset of renal replacement therapy (RRT).

**Methods:**

We conducted a population-based, retrospective case-control study of patients who developed ESRD and started RRT. We conducted the study in a health maintenance organization, Kaiser Permanente Northwest (KPNW). The case-control study was nested within the adult population of KPNW members who were enrolled during 1999, the baseline period. Cases and their matched controls were identified from January 2000 through December 2004. We evaluated baseline clinical characteristics measured during routine care by calculating the adjusted odds ratios and their 95% confidence intervals after controlling for matching characteristics: age, sex, and year.

**Results:**

The rate of RRT in the cohort from which we sampled was 58 per 100,000 person-years (95% CI, 53 to 64). After excluding patients with missing data, we analyzed 350 cases and 2,114 controls. We identified the following characteristics that predicted ESRD with odds ratios ≥ 2.0: eGFR<60 mL/min/1.73 m^2 ^(OR = 20.5; 95% CI, 11.2 to 37.3), positive test for proteinuria (OR = 5.0; 95% CI, 3.5 to 7.1), hypertension (OR = 4.5; 95% CI, 2.5 to 8.0), gout/positive test for uric acid (OR = 2.5; 95% CI, 1.8 to 3.5), peripheral vascular disease (OR = 2.2; 95% CI, 1.4 to 3.6), congestive heart failure (OR = 2.1; 95% CI, 1.4 to 3.3), and diabetes (OR = 2.1; 95% CI, 1.5 to 2.9).

**Conclusions:**

The clinical characteristics needed to predict ESRD--for example, to develop a population-based, prognostic risk score--were often documented during routine care years before patients developed ESRD and required RRT.

## Background

The National Kidney Foundation's (NKF) Kidney Disease Outcomes Quality Initiative (K/DOQI) encourages providers to conduct laboratory screening of patients to identify unrecognized chronic kidney disease (CKD) and stratify patients into stages of CKD based on their serum creatinine values--as an estimate of their glomerular filtration rate (GFR)--so that providers can intervene to slow the progression to end-stage renal disease (ESRD) and other outcomes, such as cardiovascular events and mortality[1]. The utility of the NKF guidelines is not limited to management of individual patients. The guidelines offer an opportunity to manage populations of patients, especially within large integrated healthcare systems. The use of patient demographic information, laboratory values, patient education, and comprehensive supportive services can maximize the impact of the NKF guidelines within a large population. Population-based CKD management may reduce the burden of CKD and prevent or delay patients' progression to renal replacement therapy (RRT).

Previous case-control studies that evaluated risk factors for progression to ESRD identified patients on the basis of elevated serum creatinine[2] or treatment with renal replacement therapy[3] and asked participants to recall their medical history with a focus on long-term analgesic use. Interviewing does not work well for detailed clinical characteristics, especially laboratory values measured years earlier.

Investigators using data from Kaiser Permanente Northern California conducted a cohort study among 177,570 patients who had participated in health screening during 1964 and 1973 to evaluate a range of potential baseline predictors of ESRD (as measured by RRT) during a 25-year follow-up[4]. Their study provides a wealth of explanatory insight into predictors, including some that have only recently been recognized, such as lower hemoglobin values and elevated serum uric acid levels. Because their characteristics (predictors) were collected according to a study protocol among consenting study volunteers, and their hazard ratios reflect an induction period of up to 25 years, decision-makers may still have *pragmatic *questions about predictors collected during routine care (without a protocol), among a broader spectrum of patients (without requiring consent for study participation), over a briefer period. The same pragmatic considerations hold for a large Japanese cohort study, which collected data during the early 1980s [5].

Two ongoing prospective, observational studies measured detailed clinical characteristics at baseline and continue to follow patients to predict a range of outcomes, including ESRD[6,7]. Both CRIC and STRIDE enrolled patients with known CKD who were identified in the clinic-based setting. However, it's unclear whether either study will be sufficiently powered to identify multivariable predictors of ESRD because those analyses depend on the number of events, not the number of patients. That may be one reason why both CRIC and STRIDE will evaluate a broad range of endpoints (e.g., surrogate measures of renal function, composite clinical endpoints, quality of life, etc).

In earlier work within Kaiser Permanente Northwest (KPNW), we conducted a cohort study restricted to patients with stage 3 or worse CKD to predict ESRD and the start of RRT[8]. While most patients who develop ESRD have CKD, some do not (i.e. patients who suffer acute kidney injury). To address this limitation of our study, as well as the limitations of the other studies cited above, we conducted a retrospective, nested case-control study by sampling within the KPNW population. Our eligibility criteria did not consider whether patients met laboratory criteria for CKD or had clinically recognized (diagnosed) CKD, so we hope the findings on predictors of RRT will be more pragmatic (than explanatory), and consequently of interest to decision-makers in usual care settings.

## Methods

### Design Overview and Objective

We conducted a population-based, retrospective case-control study of patients who presented with ESRD for the first time (i.e., incident cases) and started RRT while they were members of KPNW. Our objective was to identify the strength and precision of clinician-recorded predictors of RRT in the population-based setting. We also calculated the population-based incidence of RRT to put the odds ratios into context for decision-makers.

### Setting

We conducted the study in a health maintenance organization, Kaiser Permanente Northwest (KPNW), which serves the Portland, Oregon and Vancouver, Washington metropolitan area. KPNW has an annual membership of approximately 450,000 people. KPNW's electronic medical record, HealthConnect, has served as the sole medical record at all clinics since January 1997. KPNW as a research setting has been described in detail elsewhere[9]. The study was reviewed and approved by KPNW's human subjects committee.

### Identification of Cases and Controls

Patients who developed ESRD and started RRT while they were members of KPNW were eligible to serve as cases. Patients were identified from January 2000 through December 2004 and we identified patients who were treated with chronic dialysis or had a kidney transplant. A nephrologist (MT) confirmed the renal replacement therapy and its start date by reviewing the text of patients' medical records.

We frequency matched controls (10 per case) on year of RRT as well as age and sex. Controls were randomly sampled from the source population using the case's index date (i.e., the same month the case patient started RRT). Patients with a previous diagnosis or treatment for ESRD were not eligible to serve as controls because they were no longer at risk of developing ESRD. We used incidence density sampling to estimate the rate ratio from the entire cohort[10].

### Index Dates and Eligibility

All patients were assigned an index date, which was either the date they started RRT or the date they were sampled to be a control. Risk factors for ESRD were only measured during the baseline period (1997 through 1999) to ensure that the information could have predicted ESRD. Both case and control patients met the following eligibility criteria:

• Celebrated their 20^th ^birthday by the index date (with no upper age limit).

• Contributed continuous membership in KPNW since January 1999 (until the index date).

• Maintained prescription drug coverage through KPNW since January 1999 (until the index date).

All patients were required to be members of KPNW during 1999; however, many patients were members as early as 1997 and 1998. The population-based incidence calculations used the same eligibility criteria.

### Data Collection

We measured the following possible predictors of RRT using the coded information in the electronic medical record and the laboratory values (as noted for each characteristic in Table 1). We divided the characteristics into three categories: 1) demographic characteristics for matching; 2) clinical history; 3) the most recent laboratory measures during the baseline period.

**Table 1:** Baseline characteristics to predict ESRD (RRT) and how they were measured using Kaiser Permanente Northwest data during the baseline period (1997-1999)

Characteristic	Data source
**Demographic characteristics for matching**	

Age	Membership file

Sex	Membership file

CLINICAL CHARACTERISTICS	

Height and weight (body mass index)	Height and weight recorded as separate fields in the medical record.

Current smoking	Visit with ICD-9 code 305.1, or recorded as separate field in electronic medical record

Hypertension	Visit with ICD-9 codes: 401.xx - 405.xx, or blood pressure values recorded as separate fields in the electronic medical record (systolic ≥140 or diastolic ≥90)

Hyperlipidemia	Visit with ICD-9 code 272.x, or laboratory values for total cholesterol (>199 mg/dL), low density lipoprotein (>129 mg/dL) or prescription for any statin

Diabetes Mellitus	Diabetes Registry

Coronary artery disease	Visit with ICD-9 codes: 410.xx - 414.xx (excluding 414.10, 414.11, 414.19), 402.01, 402.11, 402.91, 404.01, 404.03, 404.11, 404.13, 404.91, 404.93, 429.4A, 429.9B, 429.9A

Congestive heart failure	Visit with ICD-9 codes: 428.0, 428.9, 402.01, 402.11,402.91, 404.01, 404.03, 404.11, 404.13, 404.91, 404.93, 429.4A, 429.9B, 429.9A, 428.1

Peripheral vascular disease	Visit with ICD-9 codes: 250.7x, 440.xx, 443.81, 443.9

Retinopathy	Visit with ICD-9 codes: 250.5, 362.1, 362.10, 362.02A, 362.02, 362.29

CKD	Visit with ICD-9 codes (referenced in the US Renal Data Systems Annual report in 2004): 016.0, 095.4, 189.0, 189.9, 223.0, 236.91, 250.4, 271.4, 274.1, 283.11, 403.x1, 404.x2, 404.x3, 440.1, 442.1, 447.3, 572.4, 580-588, 591, 642.1, 646.2, 753.12-753.17, 753.19, 753.2, 794.4

Kidney stones	Visit with ICD-9 codes: 592.xx, 274.11

Uric acid/Gout	Visit with ICD-9 code 274.0, or laboratory value for uric acid (>7.0 mg/dL)

**Laboratory characteristics**	

Renal function	Laboratory values for serum creatinine (glomerular filtration rate estimated from the four-variable MDRD Study equation, but omitting race and assuming everyone was white)

Inflammation	Laboratory values for sedimentation rate(> 20 mm/hr), C-reactive protein (> 0.9 mg/dL) (low serum albumin, wbc count × 2 measures, wbc without bandemia or no eosinophilia, d-dimer, inflammation codes)

Urine Protein	Laboratory values for proteinuria (1+ or greater, with negative leucocyte esterase)

Fasting plasma glucose	Laboratory values for fasting glucose (>125 mg/dL)

### Sample Size Considerations

The sample size was fixed because of the retrospective design. We observed 485 RRT starts during the period from 2000 through 2004. The effective sample size for multivariable analyses was reduced from 485 to 350 events because of missing data. One statistical approach for considering the adequacy of the sample size for predictive modelling is to consider the total number of events in relation to the number of candidate predictor characteristics and their degrees of freedom[11]. Experts recommend approximately 10 to 20 events per degree of freedom. Consequently, 350 events allowed us to consider 18 to 35 degrees of freedom.

### Statistical Analysis

Our objective was to identify the strength and precision of characteristics that predict RRT. Consequently, we modeled the data using predictive methods instead of explanatory methods[12]. We appreciate that randomly allocating patients to a characteristic wouldn't necessarily cause a higher rate of RRT. We analyzed the data using logistic regression to calculate the odds ratios and 95% confidence intervals after controlling for matching characteristics: age, sex, and calendar time.

To select characteristics for evaluation in the logistic regression model, we started with clinical characteristics that are frequently and reliably measured in routine clinical practice (e.g., hypertension). We proceeded to characteristics that may be less reliably measured (i.e., greater measurement error in the ICD-9-CM coded electronic medical record), but which are probably strong predictors of the outcome (e.g., history of clinically recognized diabetes). We retained characteristics in the equations if they were statistically significant (*P *< 0.05).

The pattern of missing data is complicated in a retrospective study where collection depends on patients' and providers' decisions to schedule outpatient visits and to measure characteristics (e.g., order a laboratory test). When investigators cannot impute missing clinical values (because they are not missing at random), one of the less biased methods is to analyze the subgroup of patients with complete data for all characteristics, the method we adopted[13].

To calculate the population-based incidence of RRT, we added the number of person-years that members were at risk of becoming RRT cases. We then divided the number of RRT cases by the total person-time at risk and calculated the exact Poisson 95% confidence interval.

## Results

Between January 2000 and December 2004, we identified 485 patients with ESRD who were treated with RRT for the first time and met all of our eligibility criteria. Ninety-six percent of the case patients had a serum creatinine measurement during the baseline period and 76% of case patients had an eGFR <60 mL/min per 1.73 m^2^. The rate of RRT in the cohort from which we sampled was 58 per 100,000 person-years (95% CI, 53 to 64) and the mean age was 66.4 years.

For most characteristics, control patients exhibited a higher frequency of missing data. For example, body mass index was missing for 7% of case patients, but 15% of control patients. Characteristics more closely associated with the diagnosis and management of kidney disease were missing more often for control patients: 32% of control patients lacked a serum creatinine value, but only 4% of case patients lacked a serum creatinine value; 49% of control patients lacked a urine dipstick measure of proteinuria, but only 27% of case patients lacked a urine dipstick measure of proteinuria. The odds ratios in Table 2 only reflect the crude predictive power of characteristics (after adjusting for the matching characteristics). Table 3 shows the distribution of characteristics that contributed to the multivariable model for the subgroup of patients with complete data and the subgroup that we excluded from the multivariable model because they were missing one or more characteristics. Missing serum creatinine values and urine protein values were the main reasons for exclusion.

**Table 2:** Baseline characteristics (1999 or earlier) evaluated for the prediction of ESRD (RRT) that occurred between 2000 through 2004; Table 1 explains how characteristics were measured.

Characteristic	% or mean cases (n = 485)	% or mean controls (n = 4,850)	Odds Ratio* (95% CI)
**Demographic matching**			
Age (mean, continuous)	64.4	64.4	(matched)
Sex (%, male is referent)	45.6	45.6	(matched)
			
**Clinical**			
BMI (%)			
<30 (referent)	51.1	52.2	
≥30	41.7	32.7	1.28 (1.06 to1.56)
Unknown	7.2	15.2	
			
Current smoking (%)			
Nonsmoker (referent)	63.9	54.0	
Smoker	32.8	33.2	0.82 (0.67 to 1.00)
Unknown	3.3	12.8	
			
Hypertension (%)	90.9	53.4	11.15 (8.03 to 15.48)
Hyperlipidemia or statin (%)	62.7	42.5	2.36 (1.93 to 2.87)
Diabetes (%)	54.0	15.9	6.67 (5.45 to 8.15)
Coronary artery disease (%)	19.8	7.9	3.07 (2.38 to 3.98)
Congestive heart failure (%)	25.0	4.4	8.25 (6.35 to 10.72)
Peripheral vascular disease (%)	23.1	3.8	8.18 (6.27 to 10.67)
Retinopathy (%)	18.1	1.1	19.41 (13.63 to 27.62)
Kidney disease (%)	53.0	1.9	61.62 (46.61 to 81.46)
Acute renal failure (%)	8.0	0.4	24.78 (13.90 to 44.18)
Kidney stone (%)	2.9	2.1	1.40 (0.79 to 2.47)
Gout/Uric acid (%)	62.7	18.3	8.20 (6.68 to 10.06)
ACEI/ARB use (%)	65.0	19.8	8.80 (7.11 to 10.89)
			
**Laboratory**			
Renal function (GFR, %)			
90+ (referent)	6.4	17.8	
60-89	13.8	38.6	1.73 (1.10 to 2.70)
<60	75.9	11.2	50.52 (32.55 to 78.42)
30-59 (Stage 3)	35.9	10.6	24.96 (15.83 to 39.36)
<30 (Stage 4 or 5)	40.0	0.6	533.51 (292.57 to 972.89)
Unknown	3.9	32.4	
			
Inflammation (%)			
No (referent)	65.0	52.7	
Yes	24.7	11.9	1.74 (1.38 to 2.19)
Unknown	10.3	35.4	
			
Urine Protein (%)			
No (referent)	28.9	47.1	
Yes	44.1	4.2	18.89 (14.31 to 24.73)
Unknown	27.0	48.7	
			
Impaired fasting plasma glucose (%)			
No (referent)	32.4	35.2	
Yes	22.5	6.8	3.64 (2.77 to 4.78)
Unknown	45.2	58.0	

* Odds ratios measure the crude or unadjusted predictions except that all characteristics are adjusted for the matching characteristics: age, sex, and year.

**Table 3:** Baseline characteristics (1999 or earlier) evaluated for the prediction of ESRD (RRT) that occurred between 2000 through 2004 according to whether patients had complete data for the logistic regression model (shown in Table 4) and the subgroup that we excluded because they were missing one or more characteristics.

Characteristic	% or mean	% or mean	% or mean	% or mean
	**Complete cases (n = 2,464)**		**Deleted from model (n = 2,871)**	

	**Cases (n = 350)**	**Controls (n = 2,114)**	**Cases** **(n = 135)**	**Controls (n = 2,736)**

**Demographic matching**				
Age (mean, continuous)	64.3	68.7	64.9	61.1
Sex (%, male is referent)	47.1	47.7	41.5	43.9
				
**Clinical**				
Hypertension (%)	94.9	65.42	80.7	44.2
Diabetes (%)	52.3	23.0	58.5	10.4
Congestive heart failure (%)	26.9	7.4	20.0	2.1
Peripheral vascular disease (%)	22.3	5.8	25.2	2.3
Gout/Uric acid (%)	72.3	30.7	37.8	8.7
				
**Laboratory**				
Renal function (GFR, %)				
90+ (referent)	4.9	25.2	10.4	12.0
60-89	12.6	57.7	17.0	23.8
<60	82.6	17.2	58.5	6.7
Unknown			14.1	57.5
				
Urine Protein (%)				
No (referent)	39.1	90.9	2.26	13.3
Yes	60.9	9.1	0.74	0.4
Unknown			97.0	86.3

The final multivariable model of statistically significant predictor characteristics included 350 case patients and 2,114 control patients (Table 4). We identified the following characteristics that predicted RRT: eGFR<60 mL/min per 1.73 m^2 ^(OR = 20.5; 95% CI, 11.2 to 37.3), positive test for proteinuria (OR = 5.0; 95% CI, 3.5 to 7.1), hypertension (OR = 4.5; 95% CI, 2.5 to 8.0), gout/positive test for uric acid (OR = 2.5; 95% CI, 1.8 to 3.5), peripheral vascular disease (OR = 2.2; 95% CI, 1.4 to 3.6), congestive heart failure (OR = 2.1; 95% CI, 1.4 to 3.3), and diabetes (OR = 2.1; 95% CI, 1.5 to 2.9). We evaluated a wide range of other characteristics that did not improve the statistical prediction of ESRD beyond the characteristics shown in Table 4. Although we presented sex-specific odds ratios for the final model, we did not test for interactions by sex.

**Table 4:** Baseline characteristics (1997-1999) evaluated for the prediction of ESRD (RRT) that occurred between 2000 through 2004 for 350 cases and 2,114 controls.

Characteristic	Odds Ratio (95% CI) men 185 cases	Odds Ratio (95% CI) women 165 cases	Odds Ratio* (95% CI) men and women 350 cases
**Demographic matching**			
Age			(matched)
Sex			(matched)
Year			(matched)
			
**Clinical**			
Hypertension	3.62 (1.65 to 7.94)	5.86 (2.47 to 13.92)	4.51 (2.53 to 8.02)
Diabetes	2.37 (1.49 to 3.77)	1.84 (1.13 to 2.98)	2.08 (1.49 to 2.90)
Congestive heart failure	2.07 (1.15 to 3.70)	2.20 (1.15 to 4.21)	2.12 (1.38 to 3.27)
Peripheral vascular disease	1.73 (0.92 to 3.27)	2.97 (1.44 to 6.12)	2.20 (1.37 to 3.55)
Gout/Uric acid	2.66 (1.62 to 4.36)	2.43 (1.50 to 3.93)	2.51 (1.78 to 3.54)
			
**Laboratory**			
Renal function (GFR)			
90+ (referent)			
60-89	1.17 (0.53 to 2.60)	2.72 (1.01 to 7.35)	1.68 (0.91 to 3.12)
<60	15.67 (7.27 to 33.80)	29.84 (11.16 to 79.79)	20.47 (11.22 to 37.34)
			
Urine Protein			
No (referent)			
Yes	5.13 (3.22 to 8.16)	4.88 2.82 to 8.47	4.99 (3.51 to 7.10)

* Odds ratios measure the multivariable or adjusted predictions, including adjustment for the matching characteristics: age, sex, and year.

## Discussion

Most observational studies that predicted ESRD using patient characteristics enrolled patients with clinically recognized CKD. We identified a broad range of clinical characteristics--many of them easily measured in the primary care setting--that could help identify patients at risk of progressing to ESRD, possibly through the development of a prognostic risk score.

Our baseline characteristics predict RRT and which patients lived long enough to start RRT. For example, among KPNW patients with stage 3 or worse CKD, the competing risk of mortality was seven times more common than RRT[8]. Although it is only one of the relevant CKD-related outcomes, our model provides a first step toward predicting which patients will start RRT in usual care; patients with those baseline characteristics may be priority candidates for clinical interventions that prevent or slow the rate of progression.

The rate of ESRD in the cohort from which we sampled cases and controls was markedly lower than the overall US rate, in part because our HMO and the metropolitan area that it serves under-represented higher-risk racial and ethnic groups compared with the entire US. Although the HMO did not collect race and ethnicity data consistently during our study period, unpublished surveys of our members have suggested that most (88%) were white, non-Hispanic. Even among white residents, the Portland metropolitan area ranked low (25^th ^percentile) based on its age- and sex-adjusted rate of ESRD (compared with the largest 25 metropolitan statistical areas in the US)[14]. Oregon and Washington belong to Network 16, a collection of five Northwestern states tracked by the US Renal Data Systems (USRDS) Annual Data Report. Network 16 states experienced the lowest age- and sex-adjusted rate of dialysis of the 18 Networks tracked by USRDS[14].

We need to consider biases that might have distorted the size of the odds ratios in this case-control study. To the extent that our final model omitted other important predictors of ESRD, the odds ratios reported here may appear larger than they would in a more complete model. Many of the characteristics were not measured systematically, especially among control patients, as revealed by the pattern of missing data: The ratio of controls to cases drops from 10 to 1 to 6 to 1 after excluding patients with missing data. Physicians ordered certain laboratory tests in part because other clinical signs raised their suspicion of CKD. For example, universal screening for proteinuria at KPNW would probably exhibit a lower odds ratio than the estimate reported in our case-control study because higher risk patients would not be tested preferentially. Our odds ratios reflect the combination of the characteristics' intrinsic predictive values and the physicians' insights for ordering the tests or recording the diagnoses. But reducing the measurement error could also strengthen the predictive power of other characteristics that were measured more systematically. For example, we only required one serum creatinine value at baseline to estimate patients' GFR, which inevitably increases the probability of false-positive classifications attributable to acute renal disease; a second serum creatinine repeated 90 or more days after the initial serum creatinine would reduce measurement error in our classification of GFR and increase the odds ratio for predicting RRT. Perneger and colleagues provide a more thorough review of potential biases in conducting epidemiologic studies of ESRD[15].

Our retrospectively measured clinical characteristics were largely consistent with the size of estimates from the most similar cohort study, conducted in patients from Kaiser Permanente Northern California. For example, our adjusted odds ratio for diabetes (OR = 2.1) was similar to the adjusted hazard ratio (HR = 2.5) reported by Hsu and colleagues even though their final model included many additional predictors and they followed patients five times longer than we did[4]. Our odds ratio for hypertension (OR = 4.5) was larger than the hazard ratio (HR = 2.9) for severe (stage 2) hypertension reported by Hsu and colleagues.

## Conclusion

The clinical characteristics needed for prediction--for example, to develop a population-based, prognostic risk score--were often documented during routine care years before patients developed ESRD and required RRT. Nephrologists have long recognized the prognostic value of many of the patient characteristics used in this case-control study. What's important for intervention in primary care is that most of the characteristics are readily available through patient history and the outpatient medical record. Future studies should develop prognostic risk scores to predict the absolute risk of developing ESRD and starting RRT, which will require a more systemic collection of laboratory findings for eGFR and proteinuria.

## Competing interests

The authors have no competing financial interest to declare because the study evaluates the natural history of chronic kidney disease and does not evaluate any products.

## Authors' contributions

EJ, DS, MT and JJ conceptualized the study and its objective. EJ and DS designed the study. MT confirmed cases of RRT by reviewing medical records. DS and MT provided clinical expertise on measuring and selecting the baseline predictor characteristics. XY extracted the data from the HMO's databases, analyzed the data statistically and contributed to the data's interpretation. EJ wrote the manuscript; DS, MT, XY and JJ revised the manuscript critically. All authors read and approved the manuscript.

## Pre-publication history

The pre-publication history for this paper can be accessed here:

http://www.biomedcentral.com/1471-2369/12/17/prepub
